# Exchange or Eliminate: The Secrets of Algal-Bacterial Relationships

**DOI:** 10.3390/plants13060829

**Published:** 2024-03-13

**Authors:** Bertille Burgunter-Delamare, Prateek Shetty, Trang Vuong, Maria Mittag

**Affiliations:** 1 Matthias Schleiden Institute of Genetics, Bioinformatics and Molecular Botany, Friedrich Schiller University Jena, 07743 Jena, Germany; prateek.shetty@uni-jena.de (P.S.); trang.vuong@uni-jena.de (T.V.); 2 Cluster of Excellence Balance of the Microverse, Friedrich Schiller University Jena, 07743 Jena, Germany

**Keywords:** antagonism, brown algae, diatoms, dinoflagellates, green algae, haptophytes, mutualism, primary metabolites, red algae, secondary metabolites

## Abstract

Algae and bacteria have co-occurred and coevolved in common habitats for hundreds of millions of years, fostering specific associations and interactions such as mutualism or antagonism. These interactions are shaped through exchanges of primary and secondary metabolites provided by one of the partners. Metabolites, such as N-sources or vitamins, can be beneficial to the partner and they may be assimilated through chemotaxis towards the partner producing these metabolites. Other metabolites, especially many natural products synthesized by bacteria, can act as toxins and damage or kill the partner. For instance, the green microalga *Chlamydomonas reinhardtii* establishes a mutualistic partnership with a *Methylobacterium*, in stark contrast to its antagonistic relationship with the toxin producing *Pseudomonas protegens*. In other cases, as with a coccolithophore haptophyte alga and a *Phaeobacter* bacterium, the same alga and bacterium can even be subject to both processes, depending on the secreted bacterial and algal metabolites. Some bacteria also influence algal morphology by producing specific metabolites and micronutrients, as is observed in some macroalgae. This review focuses on algal-bacterial interactions with micro- and macroalgal models from marine, freshwater, and terrestrial environments and summarizes the advances in the field. It also highlights the effects of temperature on these interactions as it is presently known.

## 1. Introduction

### 1.1. Definition, Phylogeny, Distribution, and Relevance of Algae

‘Algae’ refers to a heterogeneous polyphyletic group of photosynthetic eukaryotes excluding land plants (Embryophyta). Characterized by the absence of typical plant organs such as leaves, stems, or roots [1,2], they are derived from primary and secondary endosymbiosis [3], which can be succeeded by higher-order—tertiary, quaternary—endosymbioses [4]. In primary endosymbiosis, a cyanobacterium was engulfed by a eukaryotic cell, while in secondary endosymbiosis, a green or red alga was engulfed by a eukaryotic cell. Algae derived by primary endosymbiosis include the green and red algae, as well as the glaucophytes [2,3,5]. Secondary endosymbionts of the green lineages comprise the Chlorachniophytes and the Euglenoids. The red lineage includes the Cryptophytes, Haptophytes, Dinoflagellates, and Stramenopiles. The Stramenopiles are the most species-rich clade, including diatoms, brown algae, xanthophytes, and other ochrophytes [2,3,5]. Through repeated endosymbiotic events, plastids spread through the eukaryotic tree of life, spanning more than 1.6 billion years of evolution [4].

Algae are also grouped into micro- and macroalgae. Their sizes can range from extremely small, such as the picoalga *Ostreococcus* (0.8 µm) [6], to giant kelps with fronds up to 60 m in length [7]. The number of algal species has been estimated to exceed one million, with most of them being microalgae [8,9]. Algae are prominent in bodies of water, such as oceans, rivers, and lakes [7,10], and are also common in terrestrial environments [11,12]. Algae can be found on snow and ice [13], and even in the desert [14].

Algae play a significant role in global annual primary production; together with cyanobacteria, they contribute to about 50% of CO_2_ fixation on Earth [15]. As primary producers, algae are at the base of food webs [16]. Algal activities also affect biogeochemical processes, as observed with the accelerated melting of the Greenland ice sheet [17]. In the ocean, they participate in nutrient [18], carbon [19,20,21], and sulfur cycles [22,23]. Macroalgae and kelps, in particular, are ecosystem engineers and habitat builders that shelter various forms of life [24,25,26].

Algae are strongly affected by climate change; along with the increased temperatures, there is a decrease in natural populations of kelp, such as *Saccharina latissima* [27,28], and there is coral reef bleaching due to the loss of the algal symbiont (see Section 3.3). Climate-driven shifts in algal-bacterial interactions were also reported over ten years [29].

Algae are used as food [30,31] and feed [32,33], as texture agents [34,35], in biomaterial [36,37], in medicine [38,39], as fertilizer in agriculture [40,41,42], in biofuel and hydrogen production [43,44,45], and in pollution control and bioremediation [46,47].

### 1.2. The Phycosphere and Algal-Microbial Interactions

The phycosphere is the region immediately surrounding an algal cell within a phytoplankton or in soil. It is enriched in organic molecules exuded by the cell into the surrounding water [48,49]. In this microenvironment, organisms interact via chemical compounds, such as signaling molecules, nutrients, toxins, morphogens, defense compounds, and quorum-sensing signals [50,51,52,53]. These infochemicals can reach significantly higher concentrations than those measured in bulk seawater, generating chemical gradients that can be detected by marine microbes [48,54].

Bacteria can detect and navigate towards favorable chemical gradients in their immediate surroundings through a process known as chemotaxis. This involves the utilization of transmembrane chemoreceptors, which enable bacteria to measure concentrations of chemicals. Subsequently, signal transduction systems process this information, allowing bacteria to precisely modulate their motility in response to perceived chemical gradients [48,55,56]. Algae, especially flagellate ones, also react to chemicals by tactic processes [57,58].

The algal host and its associated microbiota are collectively called holobiont [25,59,60,61], and biotic interactions within such a system are drivers of algal evolution [62]. Host-microbe interactions are grouped into “positive”, “neutral”, and “negative” interactions, called mutualism, commensalism, and antagonism, respectively [63,64,65].

**Mutualism** refers to a symbiotic relationship between bacteria and microalgae, in which both organisms gain advantages, irrespective of which partner receives greater benefits. This natural symbiosis typically evolves gradually over an extended period, remaining relatively stable across several generations [66].

**Commensalism** describes a relationship in which only one symbiotic partner benefits, while the other is unaffected. Algal-bacterial associations can transition between mutualism and commensalism, depending on environmental factors [67].

A relationship marked by **antagonism** occurs when one partner adversely affects or harms the other. Presently, limited research is dedicated to the algal antagonism on bacterial partners. One bulk of research studies focuses on the detrimental effects of extracellular degrading enzymes produced by interacting bacteria, which induces lysis of algal cell walls [68,69], but knowledge about algicidal bacteria that use secondary metabolites as toxins also emerges (e.g., [70,71]).

**Figure 1:**
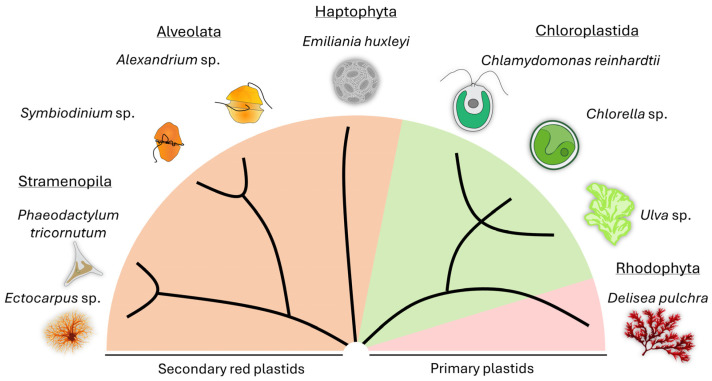
Representative micro- and macroalgal models used for studying algal-bacterial interactions are highlighted, along with their phylogenetic background. A simplified cladogram scheme derived from a phylogenetic tree [84] was taken as a basis.

Partners of algae include viruses [72,73,74,75] and bacteria [12,25,76], as well as eukaryotic organisms such as fungi [77,78,79,80] or even sloths [81]. For this review, we will focus on the bacterial partners of algae. While algae and bacteria have coexisted since the early stages of evolution, we still have limited knowledge about their detailed modes of interaction. Here, we have selected exemplary model species of micro- and macroalgae from terrestrial, freshwater, and marine environments and will present the current knowledge about their relationships with bacteria (Figure 1). We are aware that more model species have emerged; however, these are beyond the scope of this review (e.g., [2,82,83]).

## 2. Terrestrial and Freshwater Microalgae

### 2.1. The Green Microalga Chlamydomonas reinhardtii Emerges as a Soil Model

For decades, the biflagellate microalga *C. reinhardtii* has been used as a model for specific biological processes, including, for example, photosynthesis, cilia formation and function, and light-driven processes [85]. The alga can be genetically manipulated, its genome is annotated, and many molecular tools and large-scale mutant libraries are available [86,87,88,89]. Moreover, genetic crosses can be easily performed [90]. Although it is usually grown in liquid culture in the laboratory, in nature, it is mostly found in inhomogeneous wet soil [91]. It was originally isolated from a potato field in Massachusetts, USA [90].

In the past years, this microalga emerged as a model for studying microbial interactions [12,63,92], including algal-bacterial relationships. Its phycosphere was comprehensively studied by cultivating *C. reinhardtii* in soil [49], and this approach allowed for the identification of the diverse bacterial partners selected by the algal host. The algal phycosphere microbiome was then compared against the rhizosphere microbiome associated with roots of *Arabidopsis thaliana*. Surprisingly, the analysis revealed a notable overlap in the microbiome between the land plant and the chlorophyte alga, despite their evolutionary distinctiveness. Both host-associated bacterial microbiomes were dominated by Proteobacteria (Pseudomonodata), along with members of the Actinobacteria (Actinomycetota), Bacteroidetes (Bacteroidota), and Firmicutes (Bacillota) phyla [49]. These data provide an important insight into the complexity of the algal rhizosphere and reveal common bacterial communities compared to land plants.

In many investigations, dual interactions of *C. reinhardtii* and a specific bacterium were studied; mutualistic, as well as antagonistic, interactions were hereby in focus, and examples for both will be presented. In mutualistic interactions, *C. reinhardtii* has been reported to offer carbon sources in exchange for bacterial metabolites [12]. Cobalamin (vitamin B_12_) is a critical metabolite required for many cellular processes [93]. Most eukaryotic algae are cobalamin auxotrophs and must receive cobalamin exogenously or through bacterial cooperation. *C. reinhardtii* requires vitamin B_12_ for the B_12_-dependent methionine synthase called METH. As *C. reinhardtii* also encodes METE, a B_12_-independent methionine synthase, wild-type algae do not necessarily require exogenous B_12_; however, *metE* mutants must obtain vitamin B_12_ [94]. A recently evolved B_12_-dependent *C. reinhardtii* strain, *metE7*, thus establishes a mutualism with some bacteria, one of them being the rhizobium *Mesorhizobium loti* [95]. Heat stress represses *METE* gene expression; consequently, vitamin B_12_ is essential for heat-treated algal cells [96]. *C. reinhardtii* becomes thermally tolerant via a mutualistic interaction with vitamin B_12_-producing bacteria such as *Ensifer meliloti*. In this context, it is also interesting that *C. reinhardtii* synthesizes mimics of *N*-acyl-1-homoserine lactones (AHL) that stimulate quorum sensing in *E. meliloti* [97]. By quorum sensing, bacteria sense cell population numbers and react to them.

In addition to vitamins, *C. reinhardtii* profits from nitrogen sources provided by N-fixing bacteria or through N-mineralizing bacteria. For example, the diazotroph *Azotobacter chroococcum* and other *Azotobacter* species establish a mutualism with *C. reinhardtii* when any C- and N-sources are omitted in the growth media (reviewed in [12]). While *C. reinhardtii* can grow on nitrate, nitrite, ammonium, and certain amino acids, it cannot metabolize proline, hydroxyproline or peptides bearing these amino acids [98]. In coculture with *Methylobacterium* spp., the algae can grow on these substrates by establishing a mutualistic relationship with the bacterium and offering glycerol. Indole-3-acetic acid (IAA, also known as auxin) is synthesized by the algal cells via an extracellular enzyme; it inhibits algal growth, while *Methylobacterium aquaticum* degrades auxin in the presence of the alga. Thus, auxin mediates the mutualism between both microorganisms [99]. Recently, it was shown that *Microbacterium forte* sp. nov. also forms a mutual relationship with *C. reinhardtii* and even promotes algal hydrogen production [100].

Besides mutualistic interactions, antagonistic interactions with bacteria also emerged for *C. reinhardtii*. Among three examined species of the genera, *Flavobacterium*, *Xanthomonas,* and *Pseudomonas*, *P. protegens* (Pf-5) turned out to act algicidal [70]. A cocktail of toxic natural products of *P. protegens* is involved in the bacterial attack on the algae (Table 1) [70,101,102,103]. They all change the algal cell morphology, or even lyse the cells (in the case of protegencin) and influence algal growth; some secondary metabolites immobilize the algal cells (Table 1). The cyclic lipopeptide orfamide A causes the strongest immobility of algal cells with the lowest IC_50_ value [102]. Orfamide A triggers an increase in cytosolic Ca^2+^ that results in the deflagellation of the algal cells and thus renders them immotile [70,103]. Remarkably, this reaction takes place within a minute. In contrast to other cyclic lipopeptides, orfamide A does not seem to cause membrane pores [70]. The action of orfamide A depends on Ca^2+^ channels of the transient receptor potential (TRP)-type, including TRP5, TRP11, TRP15 (also known as ADF-1), and TRP22 [104]. For its activity, the N-terminal amino acids of the linear part and the terminal fatty acid tail of orfamide A are highly important [104]. One of the most potent toxins of *P. protegens* is the polyyne protegencin [101]. Protegencin acts within several hours; it blinds the cells by destroying their eyespot and lyses them [101]. This rather unstable toxin could be identified by Raman microspectroscopy, as such triple-bond-containing compounds appear in the silent region of the Raman spectrum [101].

Recently, another algicidal bacterium, *Paenibacillus polymyxa* MEZ6, was detected in soil samples. It acts against *C. reinhardtii* and other microalgae, including *Haematococcus pluvialis* and *Chlorella ellipsoidea* [107]. In this context, it should also be mentioned that orfamide A from *P. protegens* deflagellates not only *C. reinhardtii* but also a marine *Chlamydomonas* sp., as well as *Haematococcus pluvialis* and *Gonium pectorale* from the Chlorophyceaea, but not selected members from the Pedinomonaceae and Euglenophyceae [70].

Another bacterium that is antagonistic against *C. reinhardtii* is *Streptomyces iranensis*, which secretes the algicidal marginolactone azalomycin F when co-cultivated with the algal cells [108]. In tripartite cultures with the fungus *Aspergillus nidulans*, the algal cells get shielded from the bacterial toxin. These data emphasize the influence of additional partners in algal-bacterial interactions. An azalomycin F resistant fungus is needed to protect the alga [71]. The alga uses an alternative strategy when no fungus is present. In this case, a specific type of palmelloid, called a gloeocapsoid, is formed [108]. It has been postulated that the margolactone-triggered formation of such cell aggregates may have contributed to the evolution of multicellular colony-forming algae such as *Eudorina* or *Volvox* [108].

### 2.2. The Freshwater Green Alga Chlorella sp. and Its Potential for Biotechnology via Bacterial Interactions

*Chlorella* is a unicellular, non-motile green alga found in freshwater environments. The genomes of several species, including *C. vulgaris* [109], *C. sorokiniana* [110,111], and *C. variablis* [112] have been completely sequenced and characterized. Unlike *Chlamydomonas*, *Chlorella* cannot reproduce sexually; however, various transformation techniques exist to simplify genetic manipulation. The first successful attempt to genetically transform *C. vulgaris* used the electroporation technique; however, the rigid cell wall of *Chlorella* can hinder the efficiency of electroporation-based transformations [113]. Thus, cellulase was used to degrade the cell walls of *C. ellipsoidea*, facilitating the exogenous DNA uptake and leading to transformation [114]. More recently, *Agrobacterium* [115] and CRISPR-Cas9 [116] systems have been effectively employed to transform *C. sorokiniana* and *C. vulgaris*.

Members of the genus *Chlorella* have found utility across a wide array of biotechnology fields, including human and animal nutrition, bioremediation, and the production of antimicrobial compounds. Thus, there has been a persistent search for bacterial partners capable of improving either the macromolecule properties of *Chlorella* or nutrient uptake by algal-bacterial consortia.

The foremost study into the *Chlorella* phycosphere identified 29 distinct bacterial isolates from non-axenic *C. sorokiniana* cultures. These bacterial strains encompassed various genera, including *Pseudomonas*, *Acinetobacter*, *Flavobacterium*, and *Bacillus* [117]. Recent studies have investigated the effect of pairwise cocultivation on algal growth and utilized xenic *Chlorella* cultures to further explore the diversity of bacteria coexisting with *Chlorella*. Cocultivation with *Ralstonia* sp. has been shown to promote the development of *C. sorokiniana* [118], while *Brevundimonas* sp. has been found to stimulate the growth of *C. ellipsoidea* [119] and *C. vulgaris* [120]. Additionally, pairwise cultivation with *Bacillus pumilus* promotes the growth of *C. vulgaris* [121]. However, the precise mechanism of growth promotion still needs to be discovered for these pairwise combinations.

One of the most well-studied associations is between *Chlorella* and *Azospirillum brasilense*. *A. brasilense* is a well-established plant growth-promoting diazotrophic bacteria, capable of increasing the growth of 113 plant species [122]. The earliest report of *A. brasilense*-induced algal growth promotion comes from a study involving the co-immobilization of *A. brasilense* and *C. vulgaris* within alginate beads. The co-immobilization increased the total number of algal cells and pigments [123]. Subsequent research confirmed that the exudation of auxin by *A. brasilense* played a crucial role in promoting the growth of *C. vulgaris* [124]. Furthermore, transcriptomic investigations identified that IAA exudation also induces the Type 6 secretion system (T6SS1) of *A. brasilense* [125]. The T6SS is a complex multi-protein machinery crucial for modulating bacterial competition, fostering symbiotic relationships, and discerning between bacteria of the same or different species. Structurally similar to a phage tail, it enables the host bacteria to translocate effector molecules and proteins into both prokaryotic and eukaryotic cells. T6SS1 plays a pivotal role in promoting attachment between *A. brasilense* and *C. sorokiniana*, forming a physical bond between the algae and the bacterium consortium [126]. Mutants lacking T6SS1 genes exhibited reduced IAA exudation, indicating a reciprocal relationship between IAA biosynthesis and the T6SS gene cluster. While the mutant *A. brasilense* could promote algal growth, it could not induce the accumulation of carbohydrates, lipids, and proteins like the wild type, suggesting that the effector molecules and IAA were essential for these processes. However, the specific effector molecules released from T6SS1, which lead to the physiological changes observed in *C. sorokiniana*, remain elusive.

Interestingly, coculturing is not essential to promote the growth of *Chlorella*, as *A. brasilense* can release a whole collection of volatile organic compounds (VOCs). When exposed to these VOCs, *C. sorokiniana* exhibited enhanced growth and accumulation of total carbohydrates, lipids, and chlorophyll. This enhancement can be attributed to the presence of known plant growth-promoting VOCs such as 2,3-butanediol and acetoin, which are released by *A. brasilense* [121].

*Chlorella* finds immense utility as a phytoremediation agent, even though the comparatively low abundance of micro-nutrients such as iron and vitamins can hinder sustained phytoremediation. Also, algae cannot secrete chelating agents to solubilize inorganic iron [127]. In contrast, heterotrophic bacteria can produce low molecular weight chelating agents or siderophores. Siderophore-mediated iron chelation enhances the formation of bioavailable iron complexes, which algae can then absorb through plasma membrane-bound ferrireductase [128]. Consequently, the introduction of siderophore-producing *Ralstonia pickettii* establishes a mutualistic relationship whereby the bacteria benefit from the photosynthetically fixed algal exopolysaccharides while reciprocally enhancing the growth and degradation of complex azo dyes by *C. sorokiniana* [129].

It is essential to highlight that not all interactions mutually benefit both partners. In 1972, Gromov and Mamkaeva first described *Vampirovibrio chlorellavorus*, a predatory cyanobacterium with a distinct evolutionary path compared to other cyanobacteria [130]. Unlike its counterparts, *V. chlorellavorus* has completely lost its photosynthetic apparatus and instead adapted into an obligate parasite of *Chlorella*. Upon attachment to the cell wall of *Chlorella*, it uses a type 4 secretion system to form a conjugative apparatus. It can then release over 100 different hydrolytic enzymes, including proteases and peptidases, to digest its prey. Subsequently, the bacterium ingests algal lysates to aid in bacterial binary fission and plasmid replication, and finally, bacterial offsprings are released from the algal host cells to continue the cycle [131].

## 3. Marine Microalgae

Phytoplankton communities can be found in the photic zones of the oceans and are characterized by the interactions of microalgae and bacteria that coevolved and shaped the planktonic microbiome [64]. Chemical mediators, such as sulfur metabolites exchanged between the algae and bacteria, play an important role [132,133,134].

### 3.1. Phaeodactylum tricornutum: A Phytoplankton Diatom Model for Microbial Interactions

Diatoms, prominent microalgae in the oceans, are a key population of phytoplankton. Bacteria have the capacity to either promote or inhibit their growth and proliferation [135,136], and diatoms can also change their metabolic profile in response to bacteria [137]. Among these microalgae, the pennate *Phaeodactylum tricornutum*, with its diverse morphotypes, alongside the centric *Thalassiosira pseudonana*, has emerged as a model for diatom research [10,138,139]. The focus here will be on *P. tricornutum,* as it was used as a model in many algal-bacterial interactions. Its genome is known [140], it can be transformed biolistically and by electroporation, and many molecular tools are available [10,141,142,143]. Ten isolates from nine different geographic locations (seashores, estuaries, rock pools, and tidal creeks) worldwide, spanning from sub-polar to tropical latitudes, have been accessioned and well-described for this diatom [144].

The effect of different bacteria on *P. tricornutum* was investigated through algal-bacterial cocultures, identifying the presence of both mutualistic and antagonistic bacteria. The algal-bacterial interactions influenced intracellular lipids, as well as extracellular metabolites [145]. Phytoplankton-derived dissolved organic matter (DOM) is dependent on the algal strain [146]. Thus, DOM from some studied photosynthetic bacteria and the diatoms *Thalassiosira* and *Phaeodactylum* revealed a high complexity and strain dependence. It was also shown that the micro-environment plays an essential role in the bacterial interaction of *Phaeodactylum.* Introducing a polymer-based porous microtiter plate increased algal cell abundance and spatially influenced algal-bacterial interactions [147]. Moreover, exometabolites from *P. tricornutum* can influence bacterial communities, as they can act as selective bacterial growth substrates [148]. Exopolysaccharides were found to represent the largest fraction of microalgal exudates. In a coculture of *P. tricornutum* and the bacterium *Pseudoalteromonas haloplanktis*, the exo-environment described by exo-monosaccharide profiles varied depending on the culture condition [149]. This bacterium can utilize diatom-released compounds [150]. Algal-derived dissolved organic carbon and nitrogen and bacterial incorporation of these remineralized compounds were studied with *P. tricornutum* and several different bacteria. These studies defined three groups: macromolecule remineralizers, macromolecule users, and small-molecule users [151]. The relevance of dissolved organic nitrogen compounds was also shown with *Rhodobacteriaceae*-type strains that interact with *P. tricornutum* and can degrade such compounds [152].

Some bacteria act algicidal on *P. tricornutum*, such as *Kordia algicida*, which releases an algicidal protease [69]. Moreover, there are also secondary metabolites that act antagonistic. For example, the release of 2-heptyl-4-quinolone from marine bacteria causes inhibition of the cytochrome b_6_f complex in *P. tricornutum* and inhibition of its respiration [153].

### 3.2. The Coccolithophore Emiliania huxleyi: An Algal Chameleon

Coccolithophore haptophyte algae such as *E. huxleyi* are key contributors to global carbon fluxes and are essential for biogeochemistry [154]. *E. huxleyi* is ecologically highly relevant; it can form large blooms that are even visible from space. The pangenome of the *E. huxleyi* reference strain CCMP 1516 and of 13 additional isolates has been sequenced [155]. A transformation method via electroporation based on a virus approach was also established [156].

**Figure 2:**
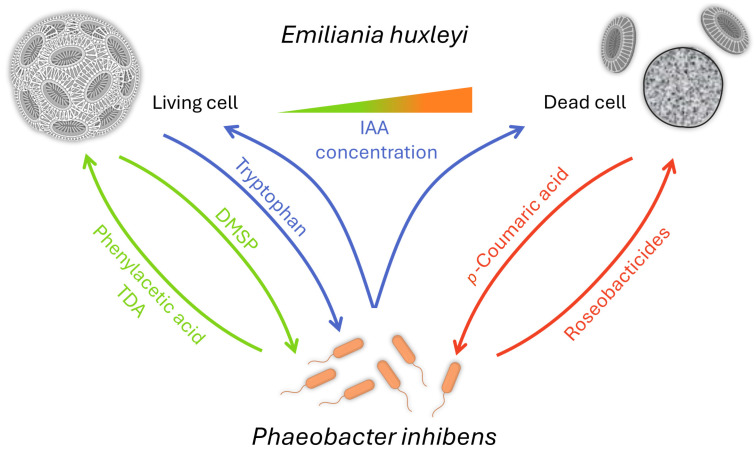
How bacterial friends turn into foes—from mutualism to antagonism [158,159,162]. *E. huxleyi* cells provide a biofilm surface and DMSP for its bacterial partner *P. inhibens* in healthy conditions. In return, the bacteria will provide the growth factor phenylacetic acid and the antibiotic TDA. In normal conditions, *E. huxleyi* cells also exude tryptophan, increasing the bacterial production of IAA and attachment to algae. When high concentrations of IAA are reached, the algal cell will enter a senescence stage and produce *p*-coumaric acid, triggering the production of the algicidal roseobactericides by *P. inhibens*.

One of the first fascinating reports dealt with the interaction of *E. huxleyi* with a Roseobacter clade bacterium named *Phaeobacter gallaeciensis*, also known as *P. inhibens* [157]. The bacterium supports algal growth via an algal growth promotor, phenylacetic acid, and an antibiotic that protects the host from bacterial pathogens called tropodithietic acid (TDA). In return, the algal cells provide dimethylsulfoniopropionate (DMSP), as C- and S-sources, and a biofilm surface to the bacteria [158,159]. It was also found that auxin plays a role in both the growth promotion of *E. huxleyi* by *P. inhibens* and the ultimate death of the algae [160]. *E. huxleyi* cells exude tryptophan in normal conditions, increasing the bacterial production of IAA and attachment to the algae. When high concentrations of IAA are reached, the algal cell will enter a senescence stage and produce *p*-coumaric acid, which initiates the synthesis of bacterial roseobacticides. These secondary metabolites are algicidal. Thus, the aging algae convert *P. inhibens* into a pathogen [158] (Figure 2). Interestingly, the synthesis of the roseobacticides is based on algal and bacterial precursor biomolecules [159]. Moreover, the genes required for TDA synthesis are also necessary for producing roseobacticides. Here, one gene cluster synthesizes two biomolecules that differ in structure and function [161].

Remarkably, associated bacterial communities can protect *E. huxleyi* from the pathogen, such as the helper bacteria *Sulfitobacter pontiacus* [163]. *P. inhibens* can also influence the production of algal alkenone lipids that play a role in estimating sea surface temperature. Unsaturated alkenones are increased in algal-bacterial cocultures, leading to changes in growth temperature up to 2 °C compared to axenic cultures [162]. A bacterial lifestyle switch with *E. huxleyi* was also observed with *Sulfitobacter* D7 [164], where algal DMSP is involved in the switch to pathogenicity. Notably, algal-produced benzoate promotes the growth of the bacterium and hinders the DMSP-caused switch [164]. The diversity of bacterial communities present in an *E. huxleyi* bloom was also studied, and the initial inoculum plays a major role in shaping the microbiome community [165]. These data emphasize that algal-bacterial relationships can transition between mutualistic and antagonistic states within the algal lifespan. Furthermore, they highlight the relevance of the surrounding bacterial microbiome as present in nature.

### 3.3. Dinoflagellate Symbiotic Symbiodiniaceae and Toxic Alexandrium sp.

Dinoflagellates are found in freshwater and oceans as a significant part of phytoplankton. They are distinguished by their enormous genome sizes, which can be attributed to the presence of hundreds of gene copies. This includes, for example, the gene encoding the luciferin-binding protein found in *Gonyaulax polyedra* [166], nowadays called *Linguludinium polyedra*. The genomic and transcriptomic characterization of a dinoflagellate genome from the toxic *Alexandrium ostenfeldii* revealed tandem repeats covering more than half of the genome [167]. Some of these microalgae are bioluminescent [168,169], and others live in symbiosis with cnidarians in reef-building corals [170]. Coral reef symbionts include mainly members from the dinoflagellate family of Symbiodiniaceae [171], whose genomes have been analyzed [172,173]. Interestingly, the associated bacterial microbiomes are distinct across symbiotic states [174].

Coral reefs are very susceptible to ocean warming, and the loss of the dinoflagellate symbiont due to climate change results in coral bleaching [170]. There is evidence that bacterial communities that are associated with Symbiodiniaceae react to heat selection and may contribute to coral adaptation to altered temperatures [175]. Increased temperature also affects the bacterial microbiome of the red tide-causing *Alexandrium minutum* [176], highlighting that global temperature increases will perturb dinoflagellate ecology significantly.

As already indicated above, some dinoflagellates, including the *Alexandrium* species, are highly toxic and dominate harmful algal blooms, also known as red tides [177]. Bacteria are usually associated with these toxic species of *Alexandrium*, such as *Pseudosulfitobacter koreense* sp. nov., together with *A. pacificum* [178] or *Haliea alexandrii* with *A. catenella* [179]. *A. catenella* produces saxitoxin and is related to shellfish poisoning, which is also highly dangerous for human consumption [180]. It was found that some marine lytic bacteria from the Proteobacteria and Cytophaga group are closely associated with this dinoflagellate. Interestingly, a change from a high-nutrient medium to a medium without organic nutrients turns them into symbiotic partners [180]. Harmful algal blooms with *A. fundyense* influence the relative abundances of bacteria and phytoplankton by repressing and promoting different taxa [181]. Microbiome stability was also investigated with an axenic strain of *A. tamarense*, to which the intact microbiome was reintroduced. Interestingly, the original microbiome was not restored; instead, a bacterial community shift to the Roseobacter clade was observed [182]. These studies show that understanding dinoflagellate-bacterial interactions is the key to understanding the biology and ecology of these fascinating, but often also highly dangerous microalgae.

## 4. Marine Macroalgae

Bacteria also colonize marine macroalgae, and these bacterial communities are not fixed and can change temporally and spatially across seasons, lifespans, life stages, and tissue types, and are shaped by biotic and abiotic factors [25,183,184]. The following section describes three macroalgal models: the red, green, and brown algae.

### 4.1. The Rhodophyceae Delisea pulchra and Its Bacterial Enemies

The red macroalga *Delisea pulchra* (Rhodophyta, Bonnemaisonales) is a widely distributed, shallow-subtidal red alga occurring throughout Southern Australia, New Zealand, Japan, and Antarctica [185,186,187]. It is one of the best-studied models for the interactions between macroalgae, bacteria, and the environment in the context of disease [188].

Bleaching disease is one of the few well-characterized examples of disease in macroalgae. A bacterial infection causes this disease [189,190], leading to the loss of photosynthetic pigments along the thallus, resulting in tissue necrosis, reduced fecundity, and increased herbivory [191]. The occurrence of bleaching disease is highly correlated with increased seawater temperatures in the summer months, which is thought to reduce the algae’s natural chemical defenses and render it more susceptible to microbial infections [192]. These infections lead to a downregulation of genes coding for predicted protein metabolism, stress response, energy generation, and photosynthesis functions. The rapid repression of genes coding for core cellular processes will likely interfere with the macroalgal antipathogen response, leading to infection, tissue damage, and bleaching symptoms [193].

Furthermore, 16S rRNA gene library analysis showed that *D. pulchra* bacterial communities contained seven phyla, including 79 species. Alpha-, Delta-, and Gammaproteobacteria are well-represented, and Planctomycetes and Bacteroidetes are relatively common in healthy *D. pulchra* [194]. Bleached *D. pulchra* microbiota is enriched in *Rhodobacteraceae*, *Saprospiraceae,* and *Flavobacteriaceae* [195]. *Colwelliaceae* and *Rhodobacteraceae* families and *Thalassomonas* and *Parvularcula* genera were the most impacted taxa between healthy and bleached communities [196]. Also, comparative metagenomics showed changes in functions associated with transcriptional regulation, cation/multidrug efflux, and nonribosomal peptide synthesis [196]. Several bacterial strains are responsible for bleaching symptoms, e.g., *Alteromonas* sp. BL110, *Aquimarina* sp. AD1 and BL5, and *Agarivorans* sp BL7 [190].

In response to the pathogen, *D. pulchra* will produce a range of secondary metabolites based on brominated furanones [189,192,197]. These molecules antagonize the same receptor as AHL—a group of widespread intercellular communication signals among bacteria. Halogenated furanones compete with and inhibit bacterial cell-to-cell communication and thus interfere with critical bacterial communication-regulated processes, such as biofilm formation and virulence [188]. They will also help the alga to shape its associated bacterial community by selecting specific groups [198]. Halogenated furanones are localized in the central vesicle of gland cells in *D. pulchra* [199]. Quantitative variations of *D. pulchra* furanones are driven by small-scale variations in environmental factors (nutrients and light) and genetic differences among individuals [200,201]. These cells release furanone onto the alga’s surface, and furanones levels on the thallus were highest near the apical tips and decreased towards the base of the alga. Variation in furanone levels within the plant and the number of gland cells followed a similar pattern [199]. These brominated furanones covalently modify and inactivate the LuxS enzyme, which produces autoinducer-2, molecules involved in bacterial quorum-sensing [202]. Furanones can also modulate the cellular concentration of the LuxR protein responsible for the reception of and response to AHLs [203,204]. In parallel to the algal chemical defense, the already established algal microbiota can prevent the pathogenicity of other bacteria, such as *Phaeobacter* sp. BS52. That bacterial strain, isolated from healthy *D. pulchra*, was antagonistic towards bleaching pathogens by significantly increasing the proportion of healthy individuals, suggesting a protective activity by preventing dysbiosis rather than direct pathogen inhibition [205].

### 4.2. The Chlorophyceae Ulva sp. Needs Its Bacteria to Get in Shape

The marine green macroalga *Ulva* (Chlorophyta, Ulvales), also named sea lettuce or gut weeds, is a model organism for morphogenesis studies, seaweed-bacteria interactions, and chemical ecology. The whole-genome sequence of *U. mutabilis* is available [206], and its genome contains genes encoding enzymes involved in the production and transformation of DMSP. A gene sequence homologous to the DMSP lyase Alma [207] enables *Ulva* to convert DMSP to dimethyl sulfide under axenic conditions [208], making this alga an essential component of the marine sulfur cycle because of its rapid growth and high DMSP content. DMSP can play multiple roles as an osmolyte and cryoprotectant in the thallus [209,210], as well as a chemoattractant [208].

In natural populations, *Ulva*-associated bacterial composition is strongly structured by both salinity and host species [211]. The bacterial communities are mainly composed of Alphaproteobacteria and Bacteroidete members, especially within the *Rhodobacteriaceae*, *Sphingomonadaceae*, *Flavobacteriaceae,* and *Sapropiraceae* families [212]. Several of these marine bacteria will have an impact on the morphologies and polymorphisms of various *Ulva* species, such as *U. clathrata* [213], *U. fasciata* [214], *U. intestinalis* [215], *U. linza* [216], *U. mutabilis* [215,217], or *U. pertusa* [218].

The first step in the interaction between *Ulva* and marine bacteria occurs during the settlement of zoospores, the motile reproductive stage of *Ulva*. Biofilms that release AHLs attract zoospores [219]. The swimming rate is reduced when zoospores detect AHLs, resulting in the accumulation of cells at the source of the AHLs. AHLs probably act as cues for the settlement of zoospores, rather than being directly involved as a signaling mechanism [50].

**Figure 3:**
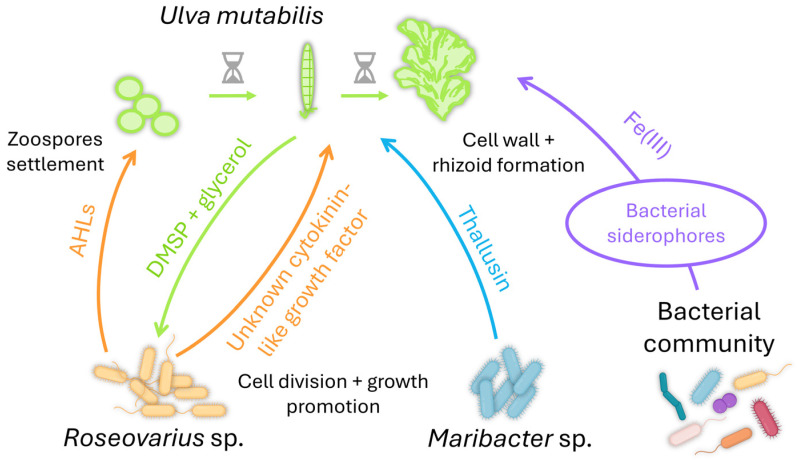
The beneficial tripartite interaction *Ulva*-*Roseovarius*-*Maribacter* (reviewed in [221]). *Roseovarius* sp. secrete AHLs that act as cues for the zoospores’ settlement. *U. mutabilis* also provides a surface enriched in glycerol and DMSP for biofilm formation by *Roseovarius* sp. This bacterial partner promotes cell division and growth by producing an unknown cytokinin-like factor. The third partner, *Maribacter* sp., induces cell wall and rhizoid formation via the production of thallusin. *Ulva* can also acquire iron from its associated bacterial communities via siderophores.

One of the most described interactions of *Ulva* with bacteria is the tripartite interaction *Ulva-Maribacter-Roseovarius* (Figure 3; [220,221]). *U. mutabilis* provides a substrate layer enriched with glycerol, a carbon source for *Roseovarius* sp., supporting biofilm formation [52]. The macroalga also releases DMSP to attract *Roseovarius* sp. [208,222], which secretes an unknown cytokinin-like growth factor promoting cell division and growth [217]. The tripartite interaction is completed with *Maribacter* sp., which is mainly found attached to the rhizoidal zone of the alga [223]. *Maribacter* sp. produces thallusin, a differentiation inducer that promotes rhizoid and cell wall formation [224]. Thallusin was isolated from the epiphytic marine bacterium YM2-23 (Cytophaga-Flavobacteria-Bacteroides group) of the green alga *Monostroma* sp. [225,226]. *Ulva* can also acquire iron from associated bacterial communities [227,228], as its genome contains iron uptake genes coding for putative transporter [206]. *Ulva* can get iron by importing a 2:1 thallusin-Fe(III)-complex [224]. The role of the Fe(III)-complex remains to be defined, as the cellular uptake of iron facilitated by thallusin derivatives was independent of their morphogenic activity [229]. These results also suggest their active import via siderophore transporters as a shuttle system.

### 4.3. The Phaeophyceae Ectocarpus sp. Needs Bacteria for Its Shape, Sex, Fitness, and Environmental Adaptation

The brown filamentous macroalga *Ectocarpus* (Ochrophyta, Ectocarpales) is a cosmopolitan genus that occurs worldwide across temperate and subtropical regions and has been sampled on all continents except Antarctica [230].

*Ectocarpus* sp. is a genetic and genomic model for brown algae [231]. The genome [232], corresponding metabolic network [233], and transformation protocol by CRISPR-Cas9 [234] are available. *Ectocarpus* is currently being used as a laboratory model to study several aspects of brown algal biology, including the implementation of in silico approaches to understand brown algal metabolism [233,235,236] and biophysical approaches to study brown algal growth and morphology [237,238]. *Ectocarpus* is also used to study interactions between brown algae and other organisms, such as symbiotic bacteria [239,240,241] or pathogens [242]. Most studies on the host-bacteria interactions in *Ectocarpus* use a screening approach by first identifying the macroalgae-associated microorganisms and characterizing potential beneficial or pathogenic effects, then creating synthetic communities and testing in vitro their effects on algal biological parameters.

It has been shown that *Ectocarpus* depends on its microbiota for its morphology, reproduction, fitness, and adaptation to a changing environment. When cultured under axenic conditions, *Ectocarpus* sp. loses its branched morphology and grows with a small ball-like appearance [240,243]. Similarly, bacterial isolates significantly promoted the production of new germlings in *Ectocarpus* [243]. Additionally, adaptation to a salinity gradient is only possible in the presence of an associated bacterial community. *Ectocarpus* relies on bacteria to facilitate the transition from low to high salinity, and cultures deprived of their associated microbiome fail to survive a transfer to freshwater. However, restoring their microflora also restores their capacity to acclimate to this change [244]. While there are compositional differences in bacterial communities associated with algal hosts, there were no functional differences, suggesting functional redundancy in the associated bacterial community [244].

The microbiota composition of lab-cultured *E. subulatus* was assessed with isolation techniques and 16S rRNA metabarcoding [239]. The identified isolates belong to 33 genera, with *Halomonas* (Gammaproteobacteria), *Bosea* (Alphaproteobacteria), and *Limnobacter* (Betaproteobacteria) being the most abundant [239]. Metabarcoding sequences data clustered into 48 operational taxonomic units. The genus *Alteromonas* represents 41.6% of the reads, making Gammaproteobacteria the most abundant class. The microbiota of *Ectocarpus subulatus* natural populations from Australia was also characterized by 16S rRNA metabarcoding [245]. The bacterial communities were dominated by Alphaproteobacteria (25% of reads), Bacteriodetes (20%), Gammaproteobacteria (8%), Planctomycetes (8%), and Actinobacteria (8%) [245].

Following the characterization of *Ectocarpus* microbiota, Karimi et al. annotated the draft genomes of seventy-two cultivable *Ectocarpus*-associated bacteria [246]. Gene clusters related to secondary metabolites production revealed that terpene, bacteriocin, nonribosomal peptide synthetases, polyketide synthases type 1 and type 3, siderophore, and homoserine lactone clusters were abundant in these genomes. Moreover, detoxification and provision of vitamin B pathways have been observed, highlighting potential contributions of bacterial metabolism toward host fitness and survival [246]. Also, coculture experiments with specific bacteria showed a significant increase in algal growth [240,241]. Seven metabolites predicted to be producible by the algae only through metabolic exchanges with specific bacteria [236,247] were selected for targeted metabolite profiling: L-histidine, putrescine, beta-alanine, nicotinic acid, folic acid, auxin, and spermidine. In several cases, these key metabolites were detected only in the inoculated cocultures [240].

## 5. Conclusions and Futures Directions

This review highlights the importance of bacteria associated with micro- and macroalgae. Bacteria can not only promote or even enable algal growth, but they are also essential for the development of some algae, shaping their morphology, as exemplified with *Ulva* and *Ectocarpus* in Section 4.2 and Section 4.3. Moreover, bacteria can be detrimental to algae, ultimately leading to their death. Often, primary and secondary metabolites are involved in these interactions. Beneficial primary metabolites include N- and C-sources or vitamins, and toxins compromise many natural products such as, for example, cyclic lipopeptides, polyynes, or phenolic compounds (see Table 1). Some metabolites are altered depending on the age of the algal culture, thus triggering the change from a mutualistic lifestyle to an antagonistic one (see Figure 2). Abiotic factors such as structured surfaces can influence microbial interactions. Moreover, temperature can play a critical role, as emphasized by coral bleaching due to the loss of the algal symbiont caused by climate change-induced heat waves. All these examples demonstrate how the bacterial microbiome associated with algae profoundly shapes their growth and life, consequently influencing their photosynthetic capacities that are necessary for sustaining life on Earth.

Currently, two major approaches are used for studying algal-microbial interactions, both with pros and cons. On one side, the entire microbiome associated with an alga is characterized, encompassing all microorganisms and their potential effects on algal life. However, such a microbiome is complex, and it is hard to understand the detailed roles of single partners and enemies in the consortium; “-omics” approaches are often used to get a first overview. Genomic and metabolomic tools are being developed to assess these interactions in silico. On the other side, individual bipartite are often examined by coculturing a single alga and a bacterium together. One alga and one bacterium are often put together in coculture. In this case, all exchanged metabolites can be studied; it can usually be predicted from which microorganism they are produced. However, this laboratory scenario does not reflect the natural environment, as we see in the case of those tripartite systems studied so far, where a third partner can already significantly change an alga’s interplay with a bacterium. In the future, it is desirable that we continuously increase the number of microorganisms in cocultures to study the influence of further partners, opportunistic pathogens, and enemies. Ultimately, we should aim to find out if new members may change their positive or negative behavior once confronted with further partners. It is also evident that the interaction of algae with bacteria is just a first step in understanding algal holobionts, as viruses, fungi, and other microorganisms will also be potential partners in the natural ecological environment.

## Figures and Tables

**Table 1:** Bacterial toxins from *P. protegens* and their effects on *C. reinhardtii*.

Toxin	Nature of Chemical Compound	Effect
Orfamide A [70,102,103]	Cyclic lipopeptide	Inhibits algal growth on plates and in liquid culture Changes cell morphology Inhibits algal motility (IC_50_ 4.1 µM) Deflagellates algal cells
Protegencin [101]	Polyyne	Inhibits algal growth in liquid culture Destroys the algal eyespot Lyses the algal cells
Pyoluteorin [102]	Natural antibiotic (hybrid stemming from a nonribosomal peptide synthase and a polyketide synthase [105])	Inhibits algal growth on plates and in liquid culture Changes cell morphology Inhibits algal motility (IC_50_ 95 µM) Deflagellates algal cells
Pyrrolnitrin [102]	Phenylpyrrole compound	Inhibits algal growth on plates and in liquid culture Changes cell morphology Inhibits algal motility (IC_50_ 20 µM) Deflagellates algal cells
Rhizoxin S2 [102]	16-membered lactone ring connected to an oxazole ring by a long unsaturated chain [106]	Inhibits algal growth on plates and in liquid culture Changes cell morphology
2,4-Diacetylphloroglucinol [102]	Phenol compound	Inhibits algal growth on plates and in liquid culture Changes cell morphology Inhibits algal motility (IC_50_ 350 µM)

## Data Availability

The used information in this review has been referenced.

## Abbreviations

AHL	*N*-acyl-homoserine lactones
DMSP	Dimethylsulfoniopropionate
DOM	Dissolved organic matter
IAA	Indole-3-acetic acid
T6SS	Type 6 secretion system
TDA	Tropodithietic acid
TRP	Transient receptor potential
VOC	Volatile organic compounds
